# Higher Education Curriculum Evaluation Method Based on Deep Learning Model

**DOI:** 10.1155/2021/9036550

**Published:** 2021-11-24

**Authors:** Mei Zuo, Jixiang Wang

**Affiliations:** ^1^Evergreen Insititute of Elderly Education, Shenzhen Polytechnic, Shenzhen 518055, China; ^2^Business School, Jinhua Polytechnic, Jinhua, Zhejiang 321000, China

## Abstract

Higher education plays an important role in the improvement of people's quality and the development of our country. Therefore, it is necessary to evaluate the higher education curriculum. This paper analyzes and constructs the deep network learning system and self-encoder and evaluates the Chongqing higher education curriculum based on the deep learning network selected by 50 universities in Chongqing. It is found that the numbers of test objects, indicators, and hidden layers have an impact on the evaluation results. At the same time, a classroom teaching model is designed to improve the quality of higher education and solve the problem of insufficient curriculum quality of higher education.

## 1. Introduction

As we all know, education has been an important issue of national concern. As the saying goes, knowledge changes destiny and science and changes the world. China has always regarded the construction of higher education as an important reform. Since the founding of the People's Republic of China, the quality of higher education has been continuously improved, and great changes have taken place, realizing the leap from quantitative change to qualitative change, so that the people of the whole country have the right to accept higher education. From the perspective of students, the researcher makes an effective and reasonable analysis of the framework and content of the traditional teaching mode. However, with the development of economy and education, traditional education can no longer meet the needs of society and students [1]. This paper puts forward the scheme of virtual reality and interactive classroom, through testing classroom learning activities and extracurricular learning activities, using special software to realize the teaching quality evaluation of virtual classroom. In existing studies, more than 1000 Norwegian 16-year-old children were investigated to evaluate their motivation, will, and deep learning style [2]. The results show that parents have no significant influence on the assessment items of the testers, while teachers have a significant influence on children's will and deep learning style. Therefore, teachers can effectively use these characteristics to correctly guide children's learning will and way. Through the analysis of deep learning model in recent research [3], the advantages, characteristics, and application occasions of SPOC model under deep learning model are understood; it is indicated that deep-learning-based model can be combined in future learning. The changes of the times make the requirements of the society constantly improve [4]; on this basis, this paper puts forward the combination of people's comprehensive development and deep learning and carries out a specific and effective research on innovative education. From the needs of personnel training, the evaluation of the current situation of deep learning, and students' cognition of themselves, this paper makes a detailed analysis step by step. From the meaning of active learning, this paper explains the importance of active learning and its value in the process of students' growth, and then, according to the attitude of the United Nations towards active learning, it summarizes that active learning has become a trend and then puts forward whether active learning can continue to be applied and then makes a detailed analysis of this problem [5]. In the meantime, some researchers analyze the general framework of the basic curriculum of higher education in four different schools on the east coast of the United States [6] and identify and depict the preevent strategies for the sustainable development of communities. The deep learning model is beneficial to the development of related courses, so it can be combined with the deep learning model in the future research of the course. Triangle theoretical model is proposed to systematically discuss the innate conditions of deep learning music education by taking professors and music teachers as the research objects [7]. The model determines the core of education through the study of the relationship among professors, trainees, and research contents. On this basis, the teaching quality is discussed in depth. Research first makes an effective and detailed analysis of the current situation and quality of higher education and then puts forward some questions about the current online teaching methods. Then, through a variety of learning methods and models given by learning experts, specific suggestions are given to improve students' deep learning [8]. Based on deep learning, research takes logistics management and engineering talents cultivation as the research object [9], puts forward “flipped learning,” and gives its concept. Finally, the model is effectively combined with logistics management engineering courses, which improves the teaching quality of higher education and promotes the progress of logistics management engineering personnel training. Sibbel et al. first make college students all over the world realize their important role in transformative learning [10] and then put forward some problems such as the sustainable development of higher education. Through the effective and high-quality research on the development activities of practical community and professionals, this paper determines the key influence of the sustainable development of education. The QUT electronic files also support the innovative and critical ability of higher education [11]. Through social practice and investigation, the electronic learning programs are explained, which play a vital role in the teaching reform of the whole school. Students are supported to have a correct understanding of the relationship between formal learning and informal learning, so that they can have a correct understanding of the relationship between learning and personal goals. A self-learning design model based on the new media environment is proposed in [12]; this model is to make higher education in Thailand more effective in cultivating students' creative thinking. The model makes a general judgment on the framework and content of the model through two survey methods. Then the teaching mode and method are determined from three aspects. The combination of network information technology and teaching to realize the deep learning of students in higher education is proposed [13]. This model uses various Internet tools, puts forward the development of network teaching mode, and puts forward the model of mixed teaching mode. Then, through the investigation and interview of students who participate in the learning of this model, the information needed to study this model is collected, and then it is found and analyzed that the most significant factors affecting the learning effect of college students are students' subjective will and teachers' input. Through questionnaires, interviews, and other ways, the data and information needed for the research are collected reasonably and efficiently, and these data and information are recorded, observed, and analyzed. Finally, the main factors influencing the loneliness of undergraduate learners are obtained, and some effective solutions are put forward [14]. The main research content of the study in [15] is the positive impact of artificial intelligence on the education industry. Firstly, it makes an effective analysis of the development of artificial intelligence and the education industry through the current situation and then understands the situation of each student and then uses the model to fit to find out the shortcomings of students; then, according to the different shortcomings of different students, teachers can better target teaching. In recent years, with the increasing popularity of machine learning algorithms like NBNs (Naive Bayesian Networks) and Extreme Gradient Boosting, they have been applied in many fields, such as sentiment analysis [16] and pattern recognition and classification [17, 18], and, at the same time, machine learning has also been applied to the field of education and achieved good results [19]. However, unlike the classic machine learning methods, deep learning based on neural networks is more popular [20]. Therefore, applying deep-learning-based method for the field of education holds great promise.

## 2. Current Situation of Higher Education

With the development of economy, China is more and more aware of the importance of higher education. Therefore, since the resumption of college entrance examination, China has focused on the development of higher education. After decades of unremitting efforts, the scale of higher education in China has been significantly expanded, and the quality of higher education is also constantly improving. Parents and people from all walks of life attach great attention to the importance of learning.

**Figure 1:**
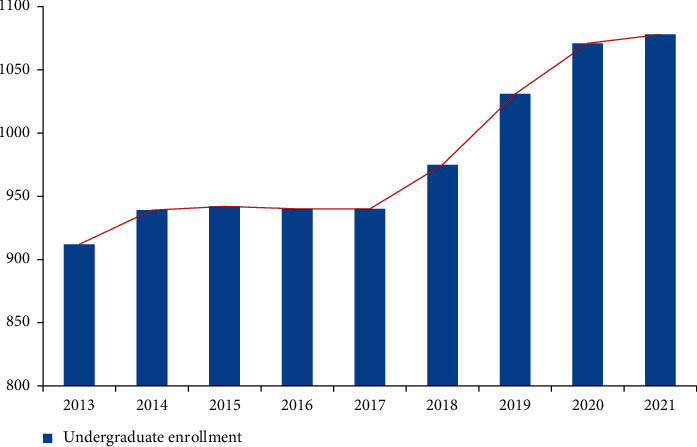
Number of candidates for college entrance examination from 2013 to 2021.

As can be seen from Figure 1, the number of candidates for the national college entrance examination in China shows an upward trend from 2013 to 2021. Specifically, from 9.12 million in 2012 to 10.78 in 2021, the number of candidates for the national college entrance examination has increased by 1.66 million in these nine years. Among them, the increase from 2018 to 2019 is the most obvious, with an increase of about 740000 people. The upward trend from 2020 to 2021 is the least obvious, with an increase of 70000 people. From 2014 to 2017, the number of candidates for the college entrance examination did not change significantly, there was no obvious upward trend, there was no obvious downward trend, and the biggest change was only 30000. The results show that, in recent years, the number of college entrance examinations in China has been increasing, which shows that the society attaches more and more importance to higher education. All people want their children to go to university and receive a deeper education. Education is the focus of our country. Through the publicity of the benefits of education and the people's personal experience, the masses of all sectors of our society have very high expectations for higher education.

**Figure 2:**
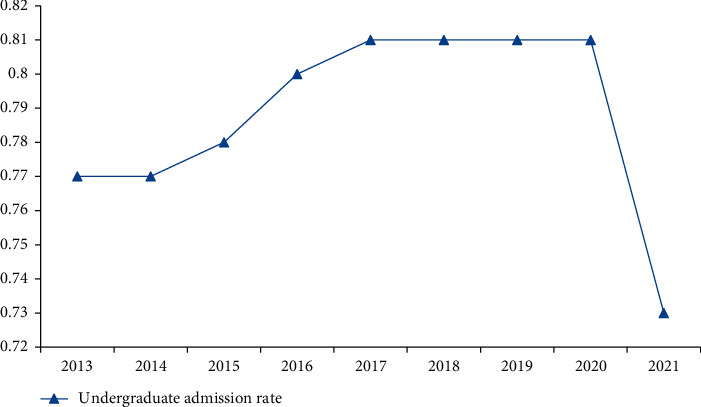
College entrance examination admission rate from 2013 to 2021.

It can be seen from Figure 2 that, from 2013 to 2021, the admission rate of college entrance examination in China first increased and then remained unchanged and finally decreased, with the largest change of 0.41% in these nine years. Since 2013, China's college entrance examination enrollment rate has been expanded, and the undergraduate enrollment rate has increased by 0.03% in five years. The admission rate of 2021 junior colleges dropped to 73%. The analysis results show that the number of people who can continue to study and go to university accounts for about 75% of the total number of applicants, which indicates that the number of people who have received higher education has always remained high, showing an overall upward trend. They also show that, with the development of economy, more and more parents and students value China's college entrance examination.

## 3. The Construction of Deep Learning Model

### 3.1. The Meaning of Deep Learning

Deep learning is proposed relative to shallow learning. Shallow learning is an early machine learning, which is simple memory and retelling. Deep learning not only meets the needs of shallow learning, but also requires students to connect individual knowledge to build a learning system, to understand knowledge while maintaining criticism and, most importantly, to apply the knowledge they have learned to practice, as shown in Table 1.

**Figure 3:**
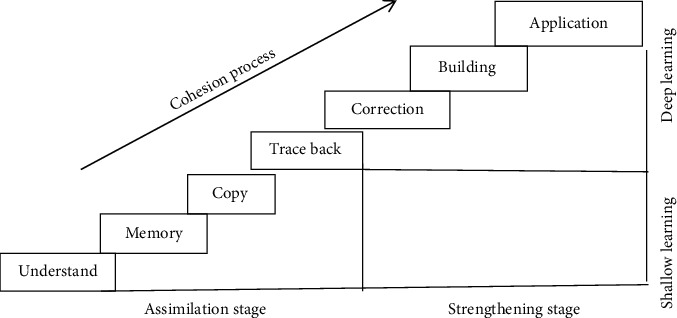
The connection between shallow learning and deep learning.

After knowing the difference between deep learning and shallow learning, this paper also makes a research and analysis on the specific process of deep learning. After consulting a large amount of data and the analysts' own experience, it can be seen that deep learning can be divided into seven processes: understanding, memory, copying, tracing, correction, building a system, and comprehensive application. These seven processes gradually transit from shallow learning to deep learning; Figure 3 shows the connection between shallow learning and deep learning.

### 3.2. The Development of Deep Learning at Home and Abroad

#### 3.2.1. Domestic Research Status of Deep Learning

**Figure 4:**
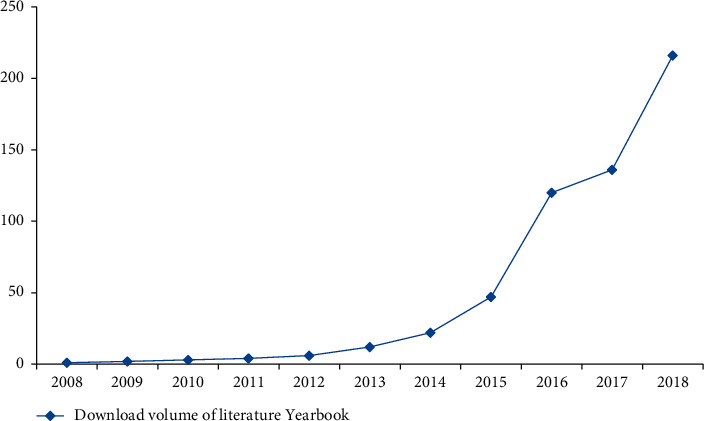
Annual download of in-depth research literature in China from 2008 to 2018.

Deep learning has been popular in China since 2010. After ten years of development, the deep learning model is more and more perfect, and the relevant literature about deep learning model is also increasing, and the download amount of the relevant literature about deep learning model is also increasing exponentially every year.  Figure 4 shows the number of annual downloads of in-depth research from 2008 to 2018.

It can be seen from the figure that the annual download volume of domestic deep learning model literature is on the rise as a whole and increases exponentially. From 2008 to 2019, a total of 215 articles have been added, with an average annual increase of 18 articles. The growth rate of annual download volume can be divided into three stages. From 2008 to 2012, there was a small increase stage, from one to four. In five years, there were only three, with an average annual increase of less than one; the second stage is from 2012 to 2016, where there was a slow increasing stage, and the overall number of articles increased from 4 to 47. In five years, the number of articles increased by 43, which is about 14 times of the first stage, with an average annual increase of 9. The third stage is the rapid increase from 2016 to 2019, from 47 articles to 216 articles, which has increased 169 articles in 4 years. The number of increased articles in the first stage is 56 times, which is 4 times of that in the second stage, with an average annual increase of 42 articles, which is equivalent to the annual download volume in 2017. The highest upsurge of deep learning model in China was from 2018 to 2019 and from 2017 to 2018. These two download volumes of annual journals increased by 80 and 73, respectively. It is obvious from the figure that, from 2017, deep learning in China began to make a sudden progress, and the literature related to deep learning model increased significantly.

#### 3.2.2. Deep Learning Abroad

The research on deep learning model abroad is a few years earlier than that in China. As the leader of deep learning model, the research on deep learning model abroad has unique views on the model. In order to understand the development of deep learning model in foreign countries, this paper introduces the deep learning model in foreign countries.

**Figure 5:**
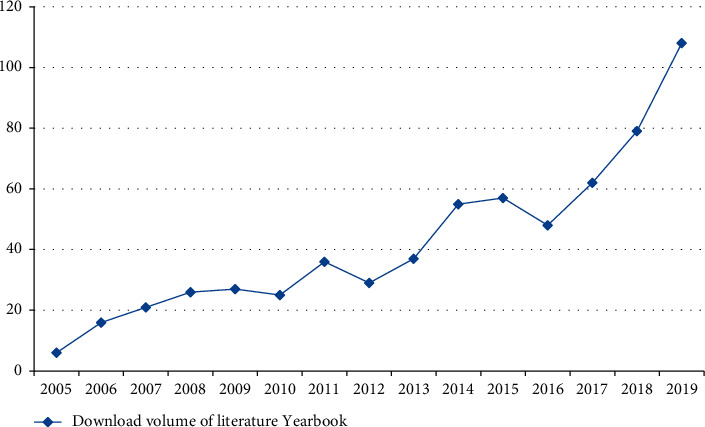
Annual download volume of in-depth research literature outside China from 2005 to 2019.

The relevant literature of the model was searched. The results are shown in Figure 5.

It can be seen from the figure that the annual download volume of foreign deep learning model literature is on the rise as a whole, with a total increase of 102 articles from 2005 to 2019, with an average annual increase of 7 articles. From 2015 to 2016, the growth rate of annual download was relatively stable, and there was no turning point of sudden increase. The highest upsurge of deep learning model in China was from 2018 to 2019, with an increase of 29 downloads. Comparing the domestic development with that of foreign countries, it is not difficult to find that although the domestic deep learning model is a few years later than that of foreign countries, the annual download increment of domestic journals in recent ten years is greater than that of foreign countries, and the increase speed is far greater than that of foreign countries, so the domestic research on the deep learning model is limited. The research is more specific and extensive than abroad.

#### 3.2.3. Instructional Design Based on Deep Learning Model

Through the previous analysis, we come to know that deep learning model can make learners learn more effectively. Therefore, we need to design an efficient and convenient teaching model, namely, the teaching design under the deep learning model.

### 3.3. Determining the Significance and Objectives of the Development of Higher Education Curriculum

**Figure 6:**
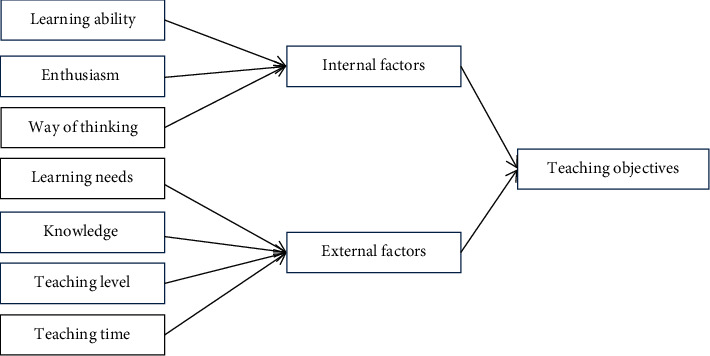
Teaching objective model.

Higher education should meet people's expectations and the development of the times. First of all, to carry out a course, we should first investigate and understand whether the course is conducive to social development and then whether it is conducive to serving the people. Curriculum objectives are divided into knowledge objectives, that is, the total amount and structure of knowledge that the course needs to impart, and student objectives, that is, the knowledge points that the course needs students to master and understand. Curriculum objectives need to be considered according to the specific situation of all aspects, combine the objectives and deep learning theory, and determine the objectives from the internal and external factors of students. External factors include teaching level, knowledge characteristics, learning needs, and teaching time. The teaching goal model is shown in Figure 6.

### 3.4. Controling and Blocking the Course Contents as a Whole

After understanding the course objectives, we need to make a general understanding of the whole course. Learners should understand the main knowledge points in each chapter and know the correlation and internal connection between chapters. This makes it convenient for students to establish the knowledge framework of the whole course. On this basis, the curriculum is divided into blocks, and the closely related chapters are divided into blocks, and the chapters with similar content structure are grouped in one block.

### 3.5. Designing the Classroom Lecture Model

The form of classroom lecture and the atmosphere of classroom are the most important links in teaching design. Good class talk learning can arouse the resonance of learners and guide them to think about problems and draw knowledge conclusions. The design of classroom lecture and the model of deep learning should be combined reasonably, taking into account the problems of classroom time, knowledge focus, learning environment, classroom interaction, bonus mechanism, lecture requirements, and so forth; in addition, the learning environment includes learning in the computer room and in the ordinary classroom. Classroom interaction includes teachers randomly selecting students to answer questions and teachers directly arranging classroom tests.

#### 3.5.1. Class Time

The length of classroom time is a direct factor that affects the content of lectures. The longer the time is, the more content of the course can be said.

#### 3.5.2. Knowledge Focus

If the time distribution of classroom lecture is affected, the key places can spend more time explaining, and the important places can be taken with them.

#### 3.5.3. Learning Environment

The important factors that affect the quality of classroom teaching and good teaching equipment can save time and make learners more able to master knowledge.

#### 3.5.4. The Mechanism of Bonus Points

The important factors of adjusting the classroom atmosphere can stimulate students' interest in learning by rewarding the usual time and other forms.

#### 3.5.5. Classroom Interaction

It can guide students to discuss and help and correct each other.

#### 3.5.6. Lecture Requirements

It is an important factor that affects the whole classroom teaching process. The classroom lectures must be explained according to the requirements, and the basic tasks of each class should be completed.

**Figure 7:**
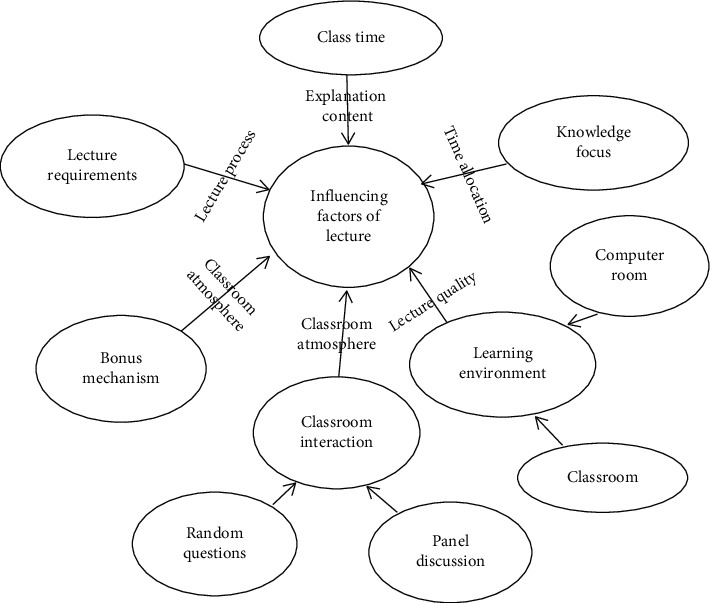
Factors influencing classroom lectures.

Classroom lecture can start with a question related to key knowledge and guide learners to discuss and think about each other; and then lectures can be given. In the process of lecture, the place requiring rich key knowledge needs to remind learners and then carry out the classroom test and explanation and arrange homework after class in Figure 7.

### 3.6. Classroom Lecture Design Evaluation Stage

**Figure 8:**
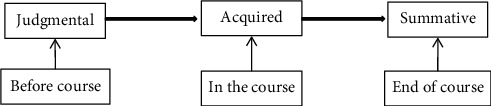
Framework of lecture design based on deep learning model.

The evaluation stage is the evaluation of the whole design model, which can detect the performance of the model. At the same time, the comprehensive evaluation is beneficial to the improvement of the design model. In the design of higher education classroom based on deep learning model, we can carry it out from three aspects: judgmental evaluation, acquisitive evaluation, and summative evaluation. Judgment line evaluation is the judgment of learners' understanding ability, thinking mode, and knowledge level. Acquisitive evaluation is the degree of learners' mastery of specific knowledge in the process of curriculum education, which is embodied in the continuous progress of learners in the learning process. Summative evaluation is a comprehensive evaluation of students' specific performance in the course of learning.  Figure 8 shows the classroom teaching evaluation model.

It can be seen from the front that the general model of classroom teaching first needs to determine the goal and significance of curriculum education.

**Figure 9:**
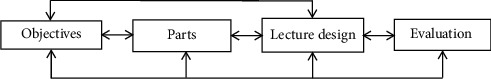
General framework of classroom teaching design.

The content of the course is divided into blocks, followed by the design of the specific content of classroom teaching and finally the evaluation and analysis of the classroom design model. These four links have a progressive relationship, they are interrelated and inseparable, and they are not simple one-way progressive, but constantly find problems in each link, and then question the previous link, and finally revise, so the whole model can be optimized in different links, so that the model can be more scientific and effective.  Figure 9 is the general framework of classroom teaching design.

## 4. Analysis of Higher Education Quality Evaluation Based on Deep Learning Model

### 4.1. Establishment of Index System

**Figure 10:**
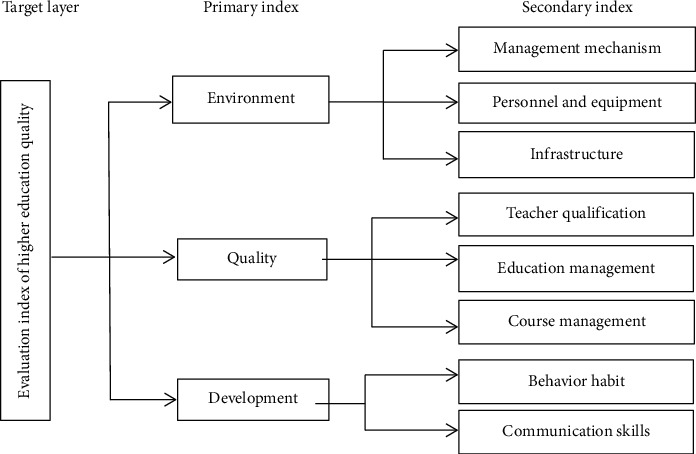
Index system of higher education.

The quality of higher education is affected by the educational environment, the quality of care and education, and the development of students. Educational environment includes management mechanism, personnel equipment, and basic equipment; the quality of care and education includes teacher qualification, education management, and curriculum management. Student development includes behavior habits and communication skills in Figure 10.

### 4.2. Evaluation Method of Deep Learning Network

**Figure 11:**
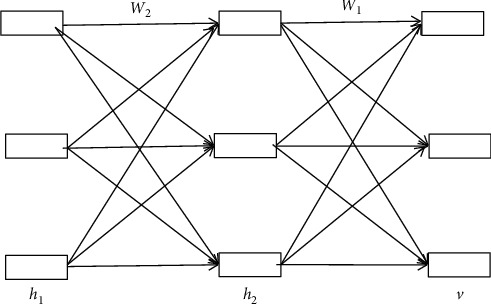
Deep information network.

Deep learning has an input layer, many hidden layers, and an output layer, and only the adjacent nodes have connections. Therefore, deep learning can be regarded as a logistic regression model. In addition, the hidden layer is not a single number but includes multiple restricted Boltzmann machines.  Figure 11 shows deep information network.

Take *v* as the visual layer, representing each secondary index *B*_*i*_ in the higher education index system, set the number of nodes in the input layer and output layer, and the number of nodes in the hidden layer is 50.

The probability distribution of visible layer and hidden layer is

(1) $$\begin{array}{c} P\left(B_{i},h_{1},h_{2}\right)=P\left(B_{i}\;|\;h_{1}\right)*P\left(B_{i}\;|\;h_{2}\right). \end{array}$$

Each layer is regarded as a restricted Bohr planter. Then the index weight of each layer is calculated:

(2) $$\begin{array}{c} P\left(V_{i}=a\;|\;h\right)=N\left(c_{i}+\sigma \sum_{j}W_{ij}h_{j}\right),\\ P\left(h_{j}=1\;|w\right)=\text{ logistic}\left(b_{j}+\sum_{i}W_{ij}\frac{vi}{\sigma }\right),\\ P\left(h_{i},B_{i}\right)=\exp\left(B_{i}^{T}Wh_{1}+c^{T}h_{1}+b^{T}A_{i}\right), \end{array}$$

where *C* is the visual layer and *B* is the hidden layer.

In the process of quality evaluation, first of all, the indicators at all levels of higher education should be abstracted, and then God's learning network should be established, respectively, which is represented by deep learning network 1 and deep learning network 2. Then the relevant tests are carried out.

Deep learning network 1 (*D*_1_) and deep learning network 2 (*D*_2_) obtained from the above steps can predict the evaluation results *qB*1test and *qB*2test of each index.

(3) $$\begin{array}{c} \left(y_{B1}^{\text{test}},q_{B1}^{\text{test}}\right)=D_{1}\left(\int_{B1}^{\text{test}}\right),\\ \left(y_{B2}^{\text{test}},q_{B2}^{\text{test}}\right)=D_{2}\left(\int_{B2}^{\text{test}}\right),\\ Q=W_{1}\cdot q_{B1}^{\text{test}}+W_{2}\cdot q_{B2}^{\text{test}}. \end{array}$$

The total weight value of the index is

(4) $$\begin{array}{c} W_{1}=\frac{{\left\|y_{B1}^{\text{test}}\right\|}_{2}^{2}}{\langle y_{B1}^{\text{test}},y_{B2}^{\text{test}}\rangle },\\ W_{2}=\frac{{\left\|y_{B2}^{\text{test}}\right\|}_{2}^{2}}{\langle y_{B1}^{\text{test}},y_{B2}^{\text{test}}\rangle }, \end{array}$$

where ‖ ‖_2_^2^ is 2-norm and 〈 〉 represents the inner product symbol.

### 4.3. Self-Encoder

**Figure 12:**
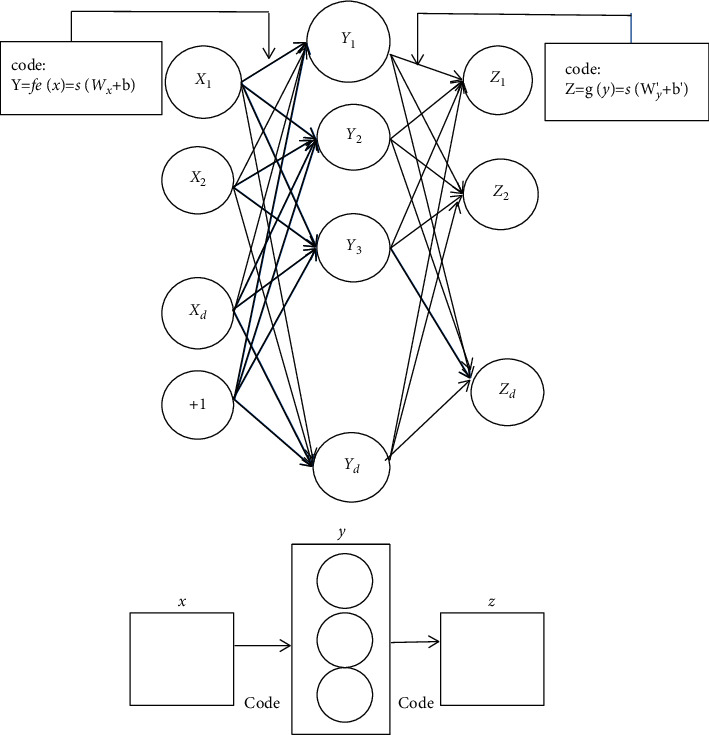
Self-encoder network structure.

Self-encoder is a kind of deep learning model, which is a kind of neural network that makes the target input equal to the output. In Figure 12, in order to construct a self-encoder network with a hidden layer, the self-encoder consists of two specific modules, encoding module and decoding module. The encoding module uses a specific mapping to map the meaning of the input vector to the representation vector of the hidden layer due to its particularity.

(5) $$\begin{array}{c} y=f_{e}\left(x\right)=s\left(W_{x}+b\right). \end{array}$$

According to the various operations of the coding segment, the information of *Y* is mapped to the decoder, and the reconstruction vector about *X* is output *z*:

(6) $$\begin{array}{c} z=g_{\theta }\left(y\right)=s\left(W{}^{\prime }y+b{}^{\prime }\right). \end{array}$$

Generally, there are *n* samples in the training sample set. Due to the mapping relationship, each sample has its corresponding potential representation vector, that is, *y* and reconstruction vector *Z*. Therefore, the parameter estimation of the model can be calculated by using these three vectors.

(7) $$\begin{array}{c} \left[\begin{array}{cc} \theta^{*} & \theta^{\prime *} \end{array}\right]=\underset{\theta ,\theta \prime }{\arg}\min\frac{1}{n}\sum_{i=1}^{n}L\left(x^{i},z^{i}\right). \end{array}$$

*L* is the loss function, which has two forms: square root error and cross entropy, two forms of formula.

(8) $$\begin{array}{c} L\left(x,z\right)={\left\|x-z\right\|}^{2},\\ L_{H}\left(x,z\right)=-\sum_{k=1}^{d}\left[x_{K}\log\;c_{k}+\left(1-x_{k}\right)\log\left(1-c_{k}\right)\right]. \end{array}$$

When there are many hidden layers, the self-coding model may not find the internal structure of the sample data set. To solve this problem, it is necessary to control the density of hidden layer cell nodes, also known as cell activation value. Calculation of unit average activation value formula is as follows:

(9) $$\begin{array}{c} \bar{y}=\frac{1}{m}\sum_{i=1}^{m}\left[y_{i}\left(x^{\left(i\right)}\right)\right]. \end{array}$$

Penalty term formula of density constraint is

(10) $$\begin{array}{c} KL\left(\rho \;\left\|\;|\bar{y}\right.\right)=\sum_{j=1}^{d{}^{\prime }}\rho \;\log\frac{\rho }{\bar{y}}+\sum_{j=1}^{d{}^{\prime }}\left(1-\rho \right)\log\frac{1-\rho }{1-{\bar{y}}_{j}}. \end{array}$$

## 5. Example Application

### 5.1. The Application of Lecture in Depth Model

Taking the university course monetary and financial statistics as an example, combined with the deep learning in this paper, the unique teaching design of this course is designed. According to the deep learning model, the course is divided into determining the course objectives, content segmentation, and classroom lecture design and model evaluation.

#### 5.1.1. Course Objectives

There are ten chapters in monetary and financial statistics, but only five chapters need to be explained according to the actual demand. They are introduction, financial assets, financial stock flow, stock and accounting principles, and monetary statistics framework. The following are the learning objectives of each chapter and the whole course (Table 2, objectives of monetary and financial statistics).

### 5.2. The Teaching Content of This Course Is Divided into Several Parts

Teaching blocks are conducive to the connection of teaching. Similar knowledge points can be taught in the same way, that is, saving time and labor costs in Table 3.

### 5.3. Lecture Design

**Figure 13:**
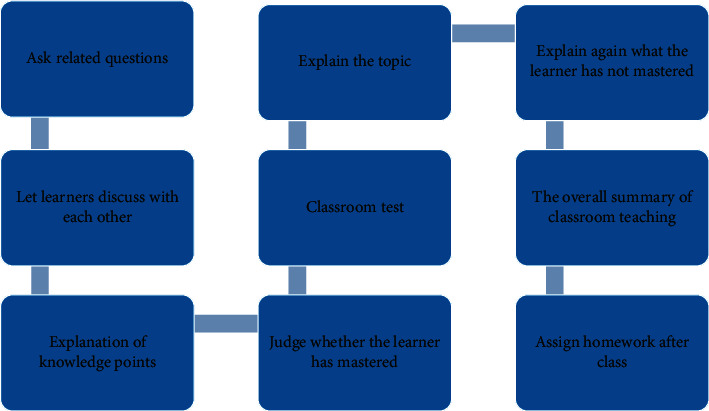
Flow chart of classroom teaching.

Before lecturing, we can inform the learners of the overall arrangement of the classroom, and then, according to the previous design of the classroom lecturing model, combined with the specific knowledge points of the course. In the process of explanation, we should observe whether the learners understand, follow the train of thought, and think seriously.  Figure 13 shows the lecture's flow chart.

### 5.4. Evaluation of Teaching Model

Teaching model evaluation can detect whether the lecturing model is reasonable, effective, and successful. In the process of continuous evaluation, the problems of lecture model are found and corrected in time. This can make the model more reasonable and more functional. Evaluation can be carried out from all aspects of the lecture model, so that the problem can be accurately modified. It can also be evaluated from the final results. The advantages of this method are direct and convenient. The following are the score of monetary and financial statistics in 2018 without the deep learning model and the score of monetary and financial statistics in 2019 with the deep learning model in Table 4.

It can be seen from Table 4 that the scores of excellent grades increased by 1.2%, the proportion of good grades increased by 6%, the proportion of medium grades increased by 11%, the proportion of passing grades remained unchanged, and the proportion of failing grades decreased by 17.4%. Therefore, we can know that deep learning model has a positive impact on the course.

In order to test the current situation of the quality of higher education in China, we take 50 colleges and universities in Chongqing as the test objects and evaluate the quality of education according to the above-mentioned evaluation model. This evaluation is divided into subjective evaluation and objective evaluation, and then the final results of subjective and objective evaluation are synthesized in a linear way. The formula is as follows:

(11) $$\begin{array}{c} X_{ij}=\frac{N\sum \xi \hat{\xi }-\sum \xi \sum \hat{\xi }}{{\left\{{\left[N\sum \xi^{2}-{\left(\sum \xi \right)}^{2}\right]}^{1/2}{\left[N\sum {\hat{\xi }}^{2}-{\left(\sum \hat{\xi }\right)}^{2}\right]}^{1/2}\right\}}^{1/2}}. \end{array}$$

The grade correlation coefficient formula is as follows:

(12) $$\begin{array}{c} C_{ij}=1-6\sum \frac{D^{2}}{N\left(N^{2}-1\right)}. \end{array}$$

#### 5.4.1. Evaluation of Index System

The index system is an important data source to evaluate the quality of higher education, which affects the quality of higher education. This experiment uses different methods to evaluate the index system of higher education reasonably, including the evaluation method based on deep learning model, the evaluation method based on analytic hierarchy process, and the evaluation method based on entropy analysis. Different results were compared.  Table 5 shows comparison of expert identification of evaluation indicators.

As can be seen from Table 5, the expert identifications of the first- and second-level indicators of deep learning model are 97.69 and 98.56, respectively, which are higher than those of AHP, 93.27 and 92.56, respectively, and higher than those of entropy analysis, 95.47 and 94.38, respectively.

#### 5.4.2. The Impact of the Number of Input Indicators on the Performance of the Model

**Figure 14:**
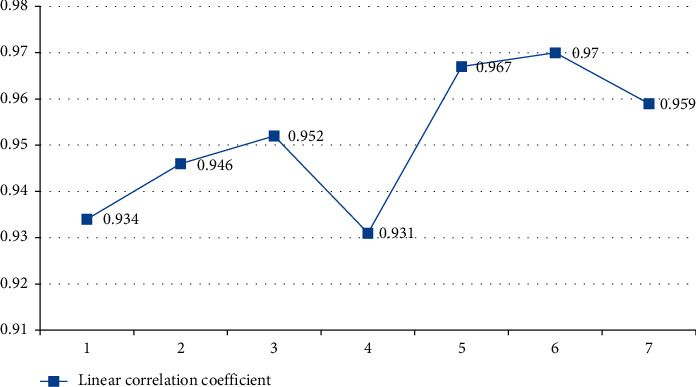
Influence of index input on linear correlation coefficient.

The more indicators are input, the less information they can provide. The following is the index input line. The influence of sex correlation coefficient is shown in Figure 14. The horizontal axis represents the number of index inputs and the vertical axis represents linear correlation coefficient, where the range is from 0 to 1.

#### 5.4.3. The Influence of the Number of Hidden Layers on Network Performance

**Figure 15:**
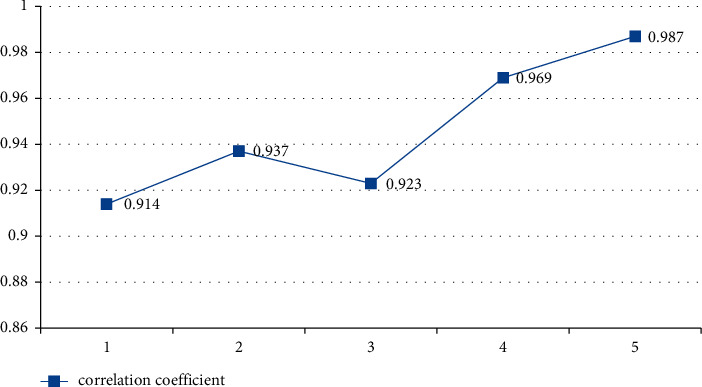
The Influence of hidden layer on deep learning function.

**Figure 16:**
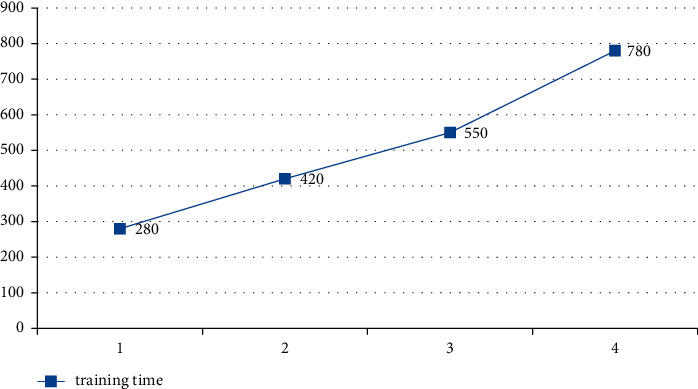
Effect of hidden layer on training time of deep learning model.

The different number of hidden layers will lead to different performance of deep learning network, as long as it will affect the correlation between the indicators and the training time of the algorithm, so the correlation coefficient and training time of the indicators with different number of hidden layers are analyzed and the training times are compared and analyzed in Figures 15 and 16. The horizontal axis of Figure 15 represents the number of hidden layers, and the vertical axis represents correlation coefficient; in Figure 16, the horizontal axis and the vertical axis represent the number of hidden layers and the training time (seconds) of the proposed model, respectively.

#### 5.4.4. The Influence of Different Test Objects on the Results

Six test objects are randomly selected from the sample, and the results of the two evaluation methods of the six test objects are obtained, which are the comparison of the text method results and the subjective evaluation results, and we obtain the linear correlation coefficients of the results of the two evaluation methods, as shown in Table 6.

**Figure 17:**
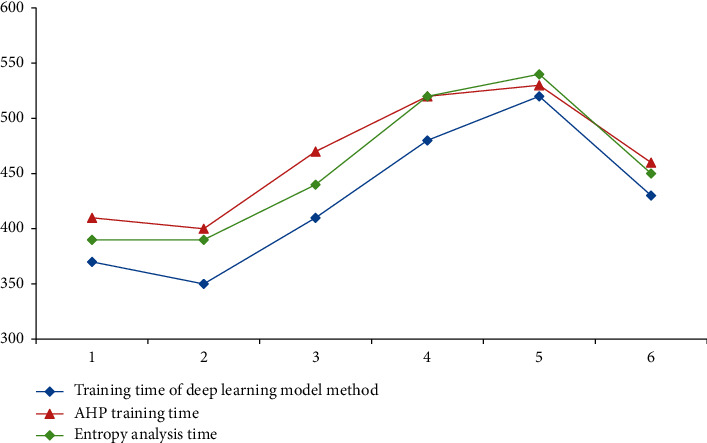
Comparison of training times of three methods under different test objects.

According to the test of different test objects and different evaluation models, we can know that the deep learning model is due to two other methods. It can be explained that the deep learning evaluation method can be used in the analysis of higher education curriculum and is also conducive to other fields of analysis in Figure 17.

It can be seen from Figure 17 that the training time of the deep learning model is significantly lower than those of the other two methods, indicating that the deep learning model can iterate more times in the same total training time, so the accuracy of the model is higher than those of the other two methods.

It can be seen from the figure that the accuracy of the analysis method of the deep learning model is significantly higher than those of the other two methods, indicating that the evaluation of higher education courses by the model is closer to reality and can better reflect the current education situation of colleges and universities in Chongqing.

**Figure 18:**
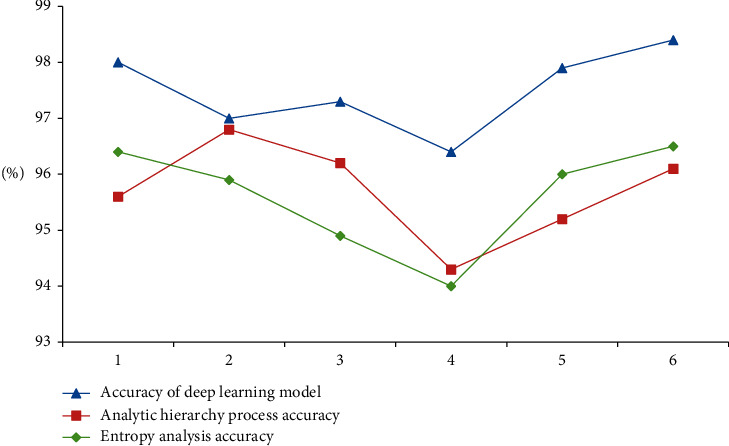
Comparison of accuracies of three methods under different test objects.

According to Table 6 and Figures 17 and 18, the in-depth learning model has high linear correlation coefficient, short training time, and the highest test accuracy. Therefore, the in-depth learning model is selected to analyze the higher education in Chongqing.

## 6. Conclusion

With the gradual popularization of higher education in China, how to evaluate the quality of higher education has become a hot research topic for scholars at present. The accumulation of more and more educational data and the development of educational technology provide the possibility for rational evaluation of higher education. In this research, we constructed the deep learning network model and self-encoder model and then evaluated the education of 50 colleges and universities in Chongqing. This evaluation only combines the deep learning network. In addition, the lecture model is constructed and applied in the course of monetary and financial statistics. Different from traditional methods such as expert rating and peer evaluation, the model based on quantitative analysis avoids the evaluation error caused by human subjectivity, making the results more objective and real and more applicable. Compared with the classic methods such as Analytic Hierarchy Process and entropy analysis method, the experimental results indicate that the proposed model has higher accuracy and shorter training time; therefore, our proposed method provides a new and effective way to evaluate the higher education in China.

## Figures and Tables

**Table 1:** Comparison of deep learning and shallow learning.

Factor	Deep learning	Shallow learning
Concept	It refers to that on the basis of understanding and memory; learners can critically and doubtfully view things and learn knowledge and then find the internal relationship between knowledge and knowledge, as well as things and things, connect individual knowledge, construct knowledge system, and finally apply the knowledge learned in real life to solve practical problems; let knowledge play its own value.	It refers to the learners who stop on the basis of recognition and understanding, can only passively learn the knowledge taught by teachers, and cannot learn new knowledge from the learned knowledge but simply copy the learned knowledge
Features	It has the characteristics of understanding and questioning, connection and system, and combination and application	It has the characteristics of being noninitiative, simple memory replication, being easy to forget, and so forth
Learning motivation	Intrinsic motivation	External motivation
Learning objectives	Requirements to improve the quality of learners; can really enhance the ability of learners	Only simple tasks and basic knowledge and skills are required
Learning view	Pay attention to the original knowledge and principle of subjective learning and knowledge innovation	Just a simple mechanical memory of knowledge and principles, with no real grasp
Learning engagement	It is positive, upward, active, and efficient learning	It is passive and inefficient learning

**Table 2:** Objectives of monetary and financial statistics course.

Chapter	Knowledge goal	Learning objectives
Chapter one	The concept of monetary statistics, the concept of financial statistics, the basic rules of monetary and financial statistics, the status and role of monetary and financial statistics.	Understand the concepts of monetary statistics and financial statistics; understand the basic rules of monetary and financial statistics; master the status and role of monetary and financial statistics.
Chapter two	The concept, classification principles, and types of financial assets.	Understand the concept of financial assets; understand classification principles and types.
Chapter three	Financial stock and flow, fixed value of financial assets and liabilities, recording time, aggregate balance, and consolidation.	Master the financial stock and flow and summarize, offset, and merge; understand the fixed value of financial assets and liabilities.
Chapter four	The concept of broad money, money and total liquidity, credit and debt.	Master the concept of broad money and the total amount of money and liquidity; understanding credit and debt.
Chapter five	Overview of monetary statistical framework, overview of central bank, overview of companies under other deposits, overview of deposit companies, overview of financial companies.	Master the overview of monetary statistics framework, central bank, deposit company, and financial company; understanding the company overview under other deposits.
Overall objectives of the course	This course requires learners to master the relevant knowledge of money and finance, such as concepts, models, and the relationship between banks and be able to reasonably combine money and finance with statistical knowledge.	

**Table 3:** Content classification of monetary and financial statistics.

Type	Course content	Teaching methods
The first category: basic concepts	The concepts of monetary statistics, financial statistics, financial assets, and generalized goods; the concept of currency	It is necessary to explain the origin, development, and different concepts of various monetary statistics, financial statistics, financial assets and generalized currencies, and so on.
The second category: statistical tools	Fixed value, recording time, aggregate balance, and financial stock of financial assets and liabilities and flow	It is necessary to deduce the methods and formulas of each statistical data, explain the principle of the formula, and test the learners.
The third category: memory and understanding	The basic rules of monetary and financial statistics; the status and role of monetary and financial statistics	After fully explaining each knowledge point, the learners are required to remember.
The fourth category: institutional framework	Overview of monetary statistical framework, overview of central bank, overview of companies under other deposits, overview of deposit companies, and overview of financial companies summary	The evolution process, the specific contents, and the framework of each framework should be explained; difference and similarities.

**Table 4:** Comparison of 2018 final score (not using model) and 2019 final score (using model).

Grade	Fraction segment	End of 2018 Number of people	End of 2018 Proportion of people (%)	End of 2019 Number of people	End of 2019 Proportion of people (%)
Excellent	95–100	4	2.2	7	3.4
Good	85–95	26	18	35	24
Secondary	75–85	31	25	42	36
Pass	60–75	27	23	34	23
Fail	<60	45	31	17	13.6

**Table 5:** Comparison of expert identifications of evaluation indicators.

Index level	Deep learning model	Analytic Hierarchy Process	Entropy analysis method
1	97.69	93.27	95.47
2	98.56	92.56	94.38

**Table 6:** Comparison of evaluation results of different test objects.

Index level	Deep learning model	Analytic Hierarchy Process	Entropy analysis method
3	0.971	0.953	0.965
3	0.971	0.953	0.965
13	0.965	0.962	0.954
7	0.984	0.947	0.949
25	0.972	0.939	0.968
39	0.966	0.955	0.958
44	0.959	0.967	0.937

## Data Availability

The experimental data used to support the findings of this study are available from the corresponding author upon request.
